# Effect of Schatzker type VI tibial plateau fractures combined with a proximal fibular and/or posterolateral joint facet fracture on early postoperative functional recovery

**DOI:** 10.1186/s13018-023-03887-2

**Published:** 2023-06-07

**Authors:** Xuezi Wang, Hu Yang, Chungui Xu, Xinzhong Xu, Chun Zhang, Juehua Jing

**Affiliations:** Department of Orthopedic Surgery, The Second Affiliated Hospital of Anhui Medical University, No. 678 Furong Road, Economic and Technological Development Zone, Hefei, 230601 China

**Keywords:** Proximal fibula, Posterolateral joint facet, Schatzker, Tibial plateau fracture

## Abstract

**Purpose:**

The objective of this study was to investigate the effect of proximal fibular and/or posterolateral joint facet (PJF) fractures on early functional recovery after Schatzker type VI tibial plateau fractures (TPFs).

**Methods:**

Seventy-nine patients with Schatzker type VI TPFs sustained from November 2016 to February 2021 were divided into three groups according to the integrity of the proximal fibula and PJF (groups A, B, and C). Details including demographics, duration of surgery, and complications were recorded. The Western Ontario and McMaster Universities Osteoarthritis index (WOMAC) score, Hospital for Special Surgery (HSS) score, lateral knee pain and lateral hamstring tightness were ascertained at the final follow-up. The HSS and WOMAC scores have high reliability in evaluating knee function and osteoarthritis.

**Results:**

There was a significant difference in the HSS score between groups A and C (*P* < 0.001) and between groups B and C (*P* = 0.036). The hospital stay was significantly different between groups A and C (*P* = 0.038) and between groups B and C (*P* = 0.013). There was a significant difference in lateral knee pain and lateral hamstring tightness between groups A and C (*P* < 0.001) and between groups B and C (*P* < 0.001).

**Conclusion:**

Our study demonstrates that proximal fibular and PJF fractures do not increase the time from injury to surgery, the incidence of complications, or the duration of surgery for Schatzker type VI TPFs. However, fractures of the proximal fibula significantly increase the hospital stay, reduce knee function, and cause lateral knee pain and lateral hamstring tightness. Combined proximal fibular fracture is more decisive than PJF involvement for prognosis.

## Introduction

A tibial plateau fracture (TPF) is a complex intraarticular fracture resulting from the combination of axial force and varus or valgus of the knee joint [1] and accounts for approximately 5–8% of lower limb fractures [2]. To restore joint stability and achieve ideal healing, surgery is recommended for patients with joint displacement or depression greater than 2 mm, condylar enlargement greater than 5 mm, poor alignment greater than 5 mm, or knee instability during movement [3, 4]. The Schatzker classification divides TPFs into types I to VI according to increasing external force, and this classification system is widely used in clinical practice [5]. Open reduction and internal fixation (ORIF) are often initially considered for the treatment of Schatzker type VI fractures and yield better results than external fixation [6]. Although internal fixation materials have become more robust and effective and surgical incisions have become less invasive, postoperative infection and traumatic osteoarthritis are still concerns.

Approximately one-third of patients with TPFs have fractures of the proximal fibula [7–9], but there have been few studies on the effect of the proximal fibula in patients with TPFs. The 2018 updated Schatzker classification did not consider the proximal fibula [10]. In recent years, the biomechanical role of the proximal fibula has been recognized. In Carrera et al.'s finite element fracture model of split collapse of the lateral tibial plateau, an intact fibula improved the axial stiffness of the model [11]. This provides a theoretical basis for early weight-bearing in patients with TPFs. Fracture of the proximal fibula also represents an injury to the posterior lateral corner (PLC). The PLC consists of the fibular collateral ligament (FCL), biceps femoris tendon (BFT), popliteal tendon, and arcuate complex [12, 13]. The PLC is a stabilizer of varus stress, external tibial rotation, and posterior translation [8]. The posterolateral support of the fibula accelerates the nonuniform settlement of the tibial plateau, which is thought to be associated with medial knee osteoarthritis [14]. However, its role in traumatic osteoarthritis of the knee is unclear. In patients with TPFs and proximal fibular fractures, lateral knee pain and lateral hamstring tightness have been found at follow-up regardless of the treatment for the proximal fibular fracture [15]. The PJF is an articular surface on the lateral condyle of the tibia that is involved in the formation of the proximal tibiofibular joint (PTFJ) and plays a role in maintaining the stability of the knee joint [12, 16]. A PJF fracture may also be one of the causes of postoperative lateral knee pain [17].

Patients with TPFs often have accompanying fractures of the proximal fibula and/or PJF. The cause of the effects of the proximal fibula, the lateral pain of the knee and the tightness of the lateral hamstring is not clear. The objective of this study was to determine whether the impact of the proximal fibula and PJF on early functional recovery after Schatzker type VI TPFs results in lateral knee pain and lateral hamstring tightness and increases the risk of traumatic osteoarthritis.

## Materials and methods

### Study design and participants

**Fig. 1:**
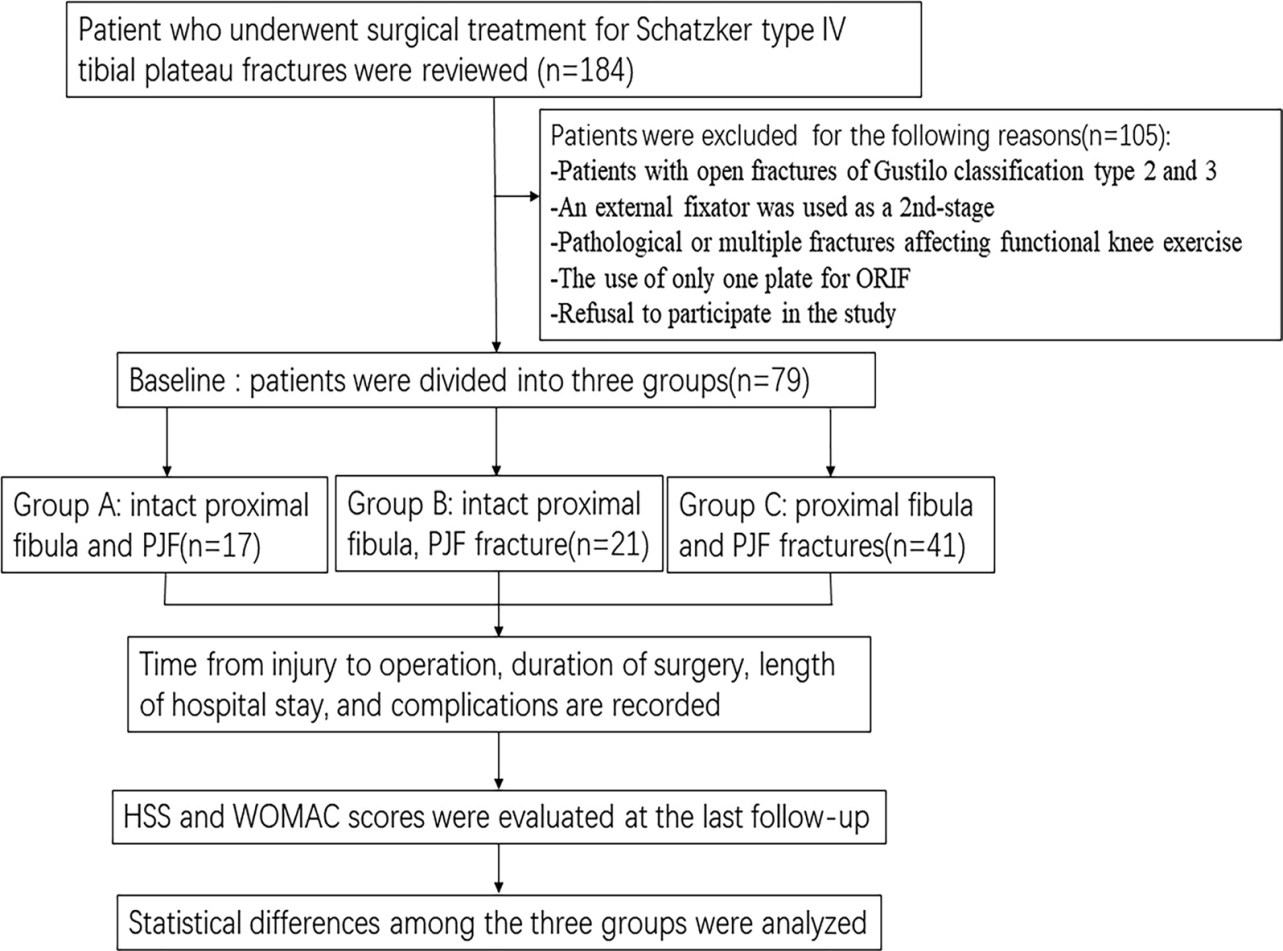
CONSORT flow diagram of patients included in this investigation. *PJF* posterolateral joint facet, *HSS* Hospital for Special Surgery, *WOMAC* Western Ontario and McMaster Universities Osteoarthritis index

The data of 184 patients who underwent surgery for Schatzker type VI TPFs from November 2016 to February 2021 were reviewed. The following inclusion criteria were used: (1) age 18 years or older, (2) all patients underwent imaging examinations before participating in this study, including X-rays and computed tomography (CT) scans, and the examination results conformed to the relevant diagnostic criteria for Schatzker type VI TPFs; (3) treatment with ORIF as a one-stage treatment, and (4) a minimum of 2 years of follow-up. The exclusion criteria were as follows: (1) patients with open fractures of Gustilo classification types 2 and 3, (2) an external fixator was used as a 2nd stage, (3) pathological or multiple fractures affecting functional knee exercise, (4) the use of only one plate for ORIF, and (5) refusal to participate in the study. Ultimately, a total of 79 patients were enrolled and divided into groups A (intact proximal fibula and PJF; 17 patients), B (intact proximal fibula and PJF fracture; 21 patients), and C (proximal fibula and PJF fractures; 41 patients). Figure 1 shows a diagram of the test design.

### Preoperative management

In all patients, preoperative radiographs and CT scans of the affected knee were obtained. Tibial skeletal traction was applied, and the limb was kept elevated on a Bohler Brown frame until the tissue oedema settled (wrinkle sign appeared) and the skin condition became good enough to post the patient for surgery. Three senior orthopedic surgeons from the same unit in our hospital (each having exposure of more than ten years) performed the surgery. Antibiotics were administered the day of surgery to prevent infection. Proximal fibular fractures were treated conservatively.

### Surgical techniques

The patient was placed in the supine position and underwent surgical treatment under general anaesthesia. A tourniquet was placed near the affected area and inflated to 260 mmHg. If significant articular surface depression was observed, autologous or allograft bone grafts were prepared.

The operative method was based on a combination of the medial and lateral approaches. Through the space between semimembranosis and the medial head of the gastrocnemius, the medial approach was carried out. Following that, the gastrocnemius muscle was retracted posteriorly and the pes anserinus tendons were retracted anteriorly. The posteromedial margin of the plateau had been visible when the semimebranosus insertion was freed at its insertion. The buttress plate was either T- or L-shaped (ZhengTian Medical Instrument Co., Ltd., TianJin, China). Depending on where the fracture fragments are located, the plate may be facing forward or backward. Using a lazy-S incision centered on the Gerdy tubercle, the lateral plate was implanted. To reveal the articular surface, the knee capsule was cut, and the meniscus was pulled upward with a holding suture. The fascia was incised and lifted with a retractor as the incision was prolonged distally while remaining at least 1 cm from the tibial crest. The anterior tibial neurovascular bundle was successfully protected within muscle during the subperiosteal dissection of the anterior tibial muscle from the lateral aspect of the proximal tibia. The depressed joint surface should be lifted and decreased with an impactor if the articular surface depression was higher than 2 mm and then used Kirschner wires or screws to fixed the broken pieces of bone. The metadiaphyseal segments and articular segment were fixed by the insertion of lateral and medial plates. Remaining bone defects were repaired using autogenous iliac bone or allogenic bone (Lianjie Biomaterials Co., Ltd., Hubei, China). Sutures were employed to fix all peripheral meniscal detachments, and if practicable, ligamentous injuries were also fixed. One drainage tube was placed on each side, and then the incision was closed. The tourniquet was released, and the surgical team checked the instruments prior to declaring the operation complete.

### Postoperative management and evaluation

All patients in the three groups were treated with the same rehabilitation regimen. Following surgery, deep vein thrombosis was avoided mechanically or chemically. Antibiotics were stopped on the first day after surgery, and the affected knee underwent passive continuous movement. On the second day after surgery, the dressing was changed, and bilateral drainage tubes were removed. The incision suture was removed after two weeks, and the time of removal was delayed appropriately for patients with possible infection. Weight-bearing was not recommended for the first 8 weeks after surgery. After 10 weeks, if the fracture was clinically and radiographically confirmed to have healed, partial weight-bearing activities were allowed and gradually increased. Anteroposterior and lateral radiographs of the affected knee were obtained at 3, 6, 18 and 24 months after surgery.

The main outcomes included functional and imaging outcomes. In general, the Western Ontario and McMaster Universities Osteoarthritis Index (WOMAC) and Hospital for Special Surgery (HSS) scores were obtained by clinical surgeons at the last follow-up to determine whether there was lateral knee pain and lateral hamstring tightness. The WOMAC score was used to evaluate the degree of arthritis, with a lower WOMAC score representing better function. The HSS score ranges from 0 to 100, with higher scores indicating better functionality. These scales have high reliability and validity [18]. Other reference results were time from injury to surgery, length of hospital stay, duration of surgery, complications, etc. The main complications were osteofascial compartment syndrome (OCS), infection, deep vein thrombosis (DVT), and common peroneal nerve injury. Infections included both superficial and deep infections; a deep infection was defined as any infection requiring surgical treatment. Deep vein ultrasound of the lower extremities was used to detect DVT. Common peroneal nerve injury was defined as weakness of the dorsal foot or toe under extension and loss of sensation of the dorsal foot [19].

### Statistical analysis

Statistical analyses were performed using IBM SPSS statistics version 25.0 software. Continuous variables are expressed as the median (interquartile spacing) and range, and categorical variables are expressed as numbers and percentages (%). If the continuous variables were normally distributed, the Welch t test of independent samples was used. For nonnormal distributions, the Kruskal‒Wallis H test was used, followed by the Holm‒Bonferroni correction. Categorical variables were compared by the chi-square test or Fisher’s test. A value of *P* < 0.05 was considered significant.

## Results

### Patient characteristics

After applying the exclusion criteria, a total of 79 of the 184 hospitalized patients met the inclusion criteria. There were 47 (59.49%) males and 32 (40.51%) females, and the mean age was 50.20 years, ranging from 18 to 80 years. Groups A, B and C included 17 (21.52%), 21 (26.58%) and 41 patients (51.90%), respectively. There were 31 (39.24%) left-sided cases and 48 (60.76%) right-sided cases. In this study, there were no significant differences in demographic data among the three groups (Table 1). Hence, they were comparable.

**Table 1:** Comparison of the patient demographic characteristics among the groups

Variable	A	B	C	*P *value
Age (years), median (IQR) (range)	45 (20) (20–70)	50 (16.50) (24–61)	53 (15.50) (18–80)	0.121
Sex (male), *n* (%)	8 (47.01)	12 (57.14)	27 (65.85)	0.401
Affected side (right), *n* (%)	8 (47.01)	11 (52.38)	12 (29.27)	0.160
Follow-up (mouths), median (IQR) (range)	36 (38) (24–81)	50 (38) (24–76)	44 (28.50) (24–78)	0.282

*IQR* interquartile spacing

### Outcome measurements

Table 2 shows that a proximal fibular fracture and/or PJF fracture had no significant effects on the time from injury to surgery, duration of surgery, WOMAC score, or incidence of complications (*P* > 0.05). Further study showed that simultaneous fractures of the proximal fibula and PJF had a significant influence on the length of hospital stay (*P* < 0.05). There were significant differences in the HSS score as well as lateral knee pain and lateral hamstring tightness in those with proximal fibular fractures (*P* < 0.05). There were no significant differences in the length of hospital stay, HSS score, or lateral knee pain and lateral hamstring tightness in the patients with PJF fractures (*P* > 0.05) (Table 3).

**Table 2:** Clinical data and follow-up data of the three groups

Variable	A	B	C	*P* value
Length of hospital stay (days), median (IQR) (range)	16 (8) (9–25)	16 (4.50) (11–31)	22 (11.50) (10–58)	0.004
Time from injury to operation (days), median (IQR) (range)	8 (4.50) (5–15)	10 (4) (5–16)	10 (6.50) (0–33)	0.057
Duration of surgery (hours), median (IQR) (range)	3.12 (2.11) (1.88–6.52)	3.83 (1.33) (1.77–5.02)	3.33 (1.68) (2–6.65)	0.235
HSS score, median (IQR) (range)	89 (11.5) (73–99)	87 (16) (41–99)	76 (12) (46–92)	< 0.001
WOMAC score, median (IQR) (range)	6 (3) (1–11)	6 (7) (1–38)	6 (6.50) (1–43)	0.770
Complications (yes), *n* (%)	1 (5.89)	3 (14.29)	11 (26.83)	0.147
DVT, *n* (%)	1 (5.89)	2 (9.52)	9 (21.95)	
Superficial infection, *n* (%)	1 (5.89)	1 (4.76)	4 (9.76)	
Deep infection, *n* (%)	0 (0)	0 (0)	3 (7.32)	
CPN injury, *n* (%)	0 (0)	0 (0)	6 (14.63)	
OCS, *n* (%)	0 (0)	0 (0)	2 (4.88)	
Lateral knee pain and lateral hamstring tightness, *n* (%)	2 (11.76)	4 (19.05)	38 (92.68)	< 0.001

*IQR* interquartile spacing, *HSS* Hospital for Special Surgery, *WOMAC* Western Ontario and McMaster University Osteoarthritis Index, *DVT* deep vein thrombosis, *CPN* common peroneal nerve, *OCS* osteofascial compartment syndrome

**Table 3:** Comparison among groups A, B and C

Variable	*P* value 1	*P* value 2	*P* value 3
Days in hospital	> 0.999	0.038	0.013
HSS score	0.429	< 0.001	0.036
Lateral knee pain and lateral hamstring tightness	0.540	< 0.001	< 0.001

1 Difference (*P* value) between groups (comparison of A vs. B); 2 Difference (*P* value) between groups (comparison of A vs. C); 3 Difference (*P* value) between groups (comparison of B vs. C)

*HSS* Hospital for Special Surgery

## Discussion

TPFs are complex intraarticular fractures that often involve ligaments and articular cartilage [20]. The objectives of the surgical management of TPFs include anatomical reconstruction of the articular surface, restoration of proper lower limb alignment, and stable fixation to maintain reduction and avoid further damage to the articular surface [10, 21, 22]. Temporary external fixation, ORIF, arthroscopic-assisted reduction and internal fixation (ARIF) and external fixators have their own advantages in treating complex TPFs [23]. The actual treatment plan depends on the surgeon's experience. Complex TPFs are usually caused by high-energy trauma, and sequelae can seriously affect the patient’s function and mental health and pose a substantial challenge for experienced surgeons [6]. Fracture morphology, trauma mechanism and soft tissue damage are key factors in determining the treatment strategy and resulting outcomes [20]. Schatzker type VI TPFs are characterized by bicondylar fractures with metaphyseal fractures, most of which result from high-energy trauma with severe peripheral soft tissue damage [24, 25], and approximately 77.4% of patients also have concomitant proximal fibular fractures [16]. Therefore, it is difficult to achieve an ideal outcome in the treatment of this type of fracture, and it is necessary to fully understand the factors that may lead to poor recovery in patients with Schatzker type VI TPFs.

**Fig. 2:**
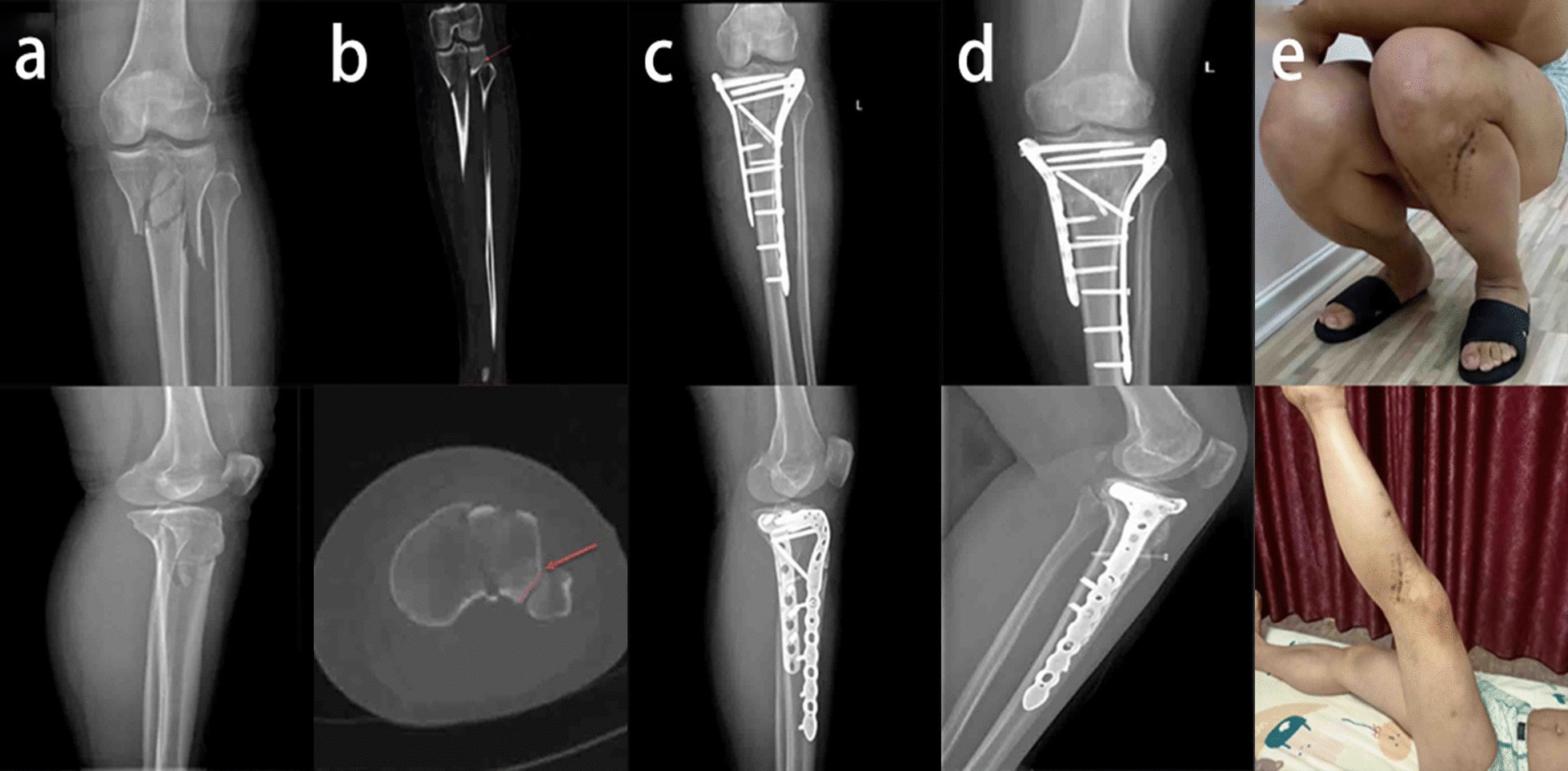
**a** Preoperative anteroposterior and lateral radiographs of a 45-year-old male patient. **b** CT scan showing that the proximal fibula and PJF (red arrow) were intact. **c** Postoperative anteroposterior and lateral radiographs showing fixation of the TPFs with double plates. **d** Anteroposterior and lateral radiographs indicating good fracture healing one year after surgery. **e** Satisfactory knee motor function and lower limb muscle strength

**Fig. 3:**
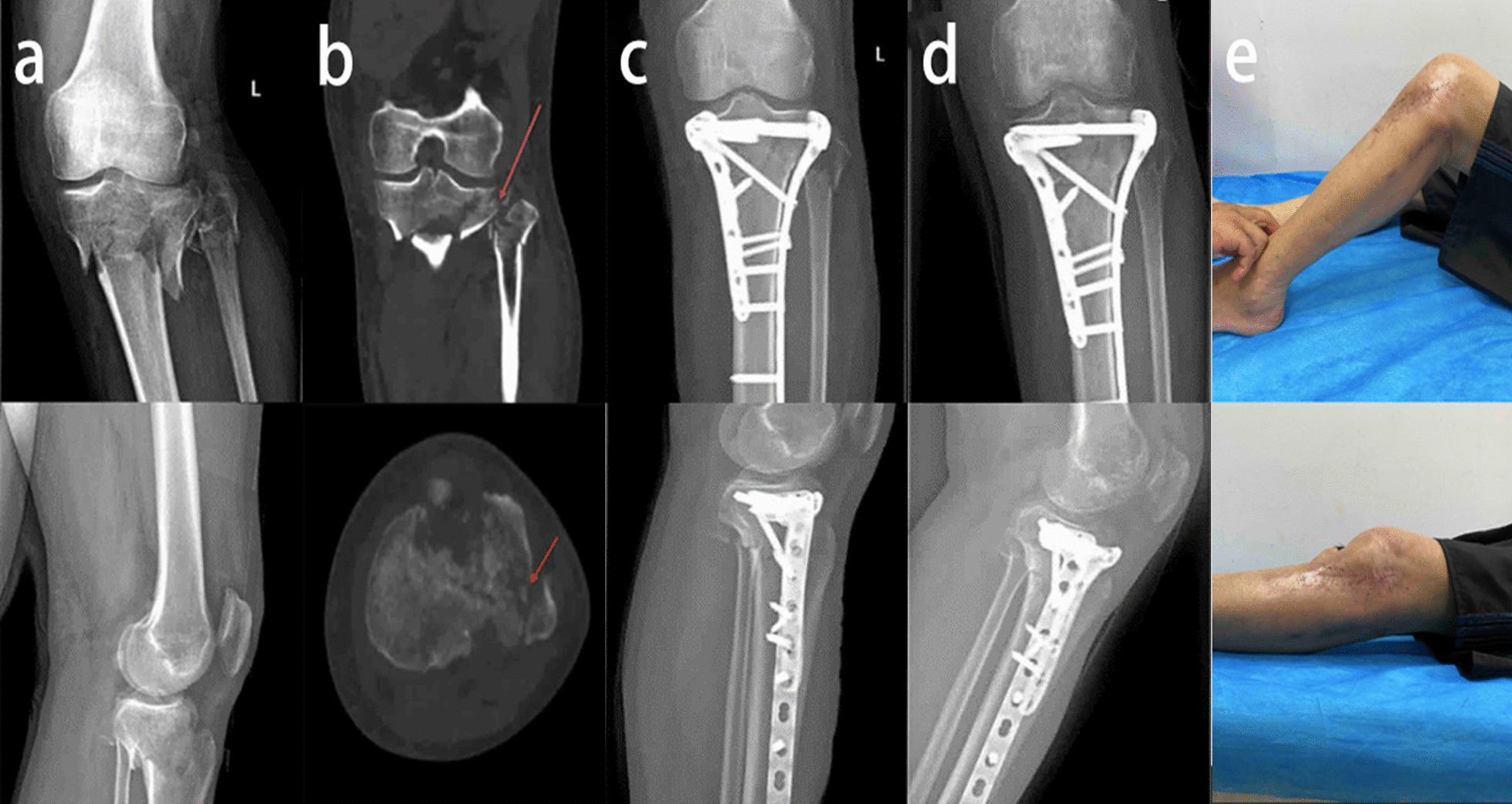
**a** Preoperative anteroposterior and lateral radiographs of a 38-year-old male patient. **b** CT scan indicating fractures of the proximal fibula and PJF (red arrow). **c** Postoperative anteroposterior and lateral radiographs. **d** Anteroposterior and lateral radiographs indicated good fracture healing six months after the operation. **e** Slightly limited flexion function

**Fig. 4:**
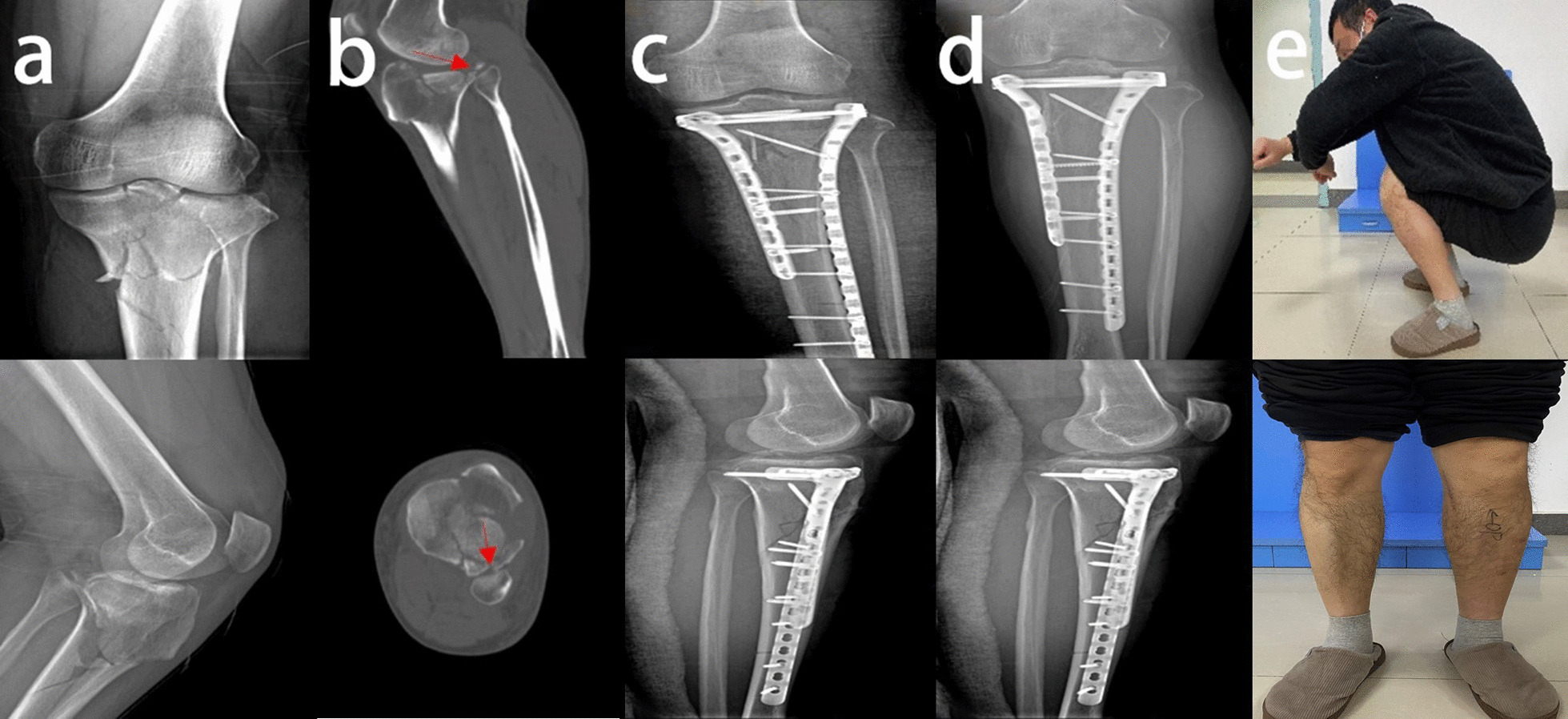
**a** Preoperative anterolateral radiographs of a 50-year-old male patient. **b** CT scan indicating PJF fracture (red arrow) with an intact proximal fibular. **c** Postoperative anterolateral radiographs. **d** Anteroposterior and lateral radiographs indicating good fracture healing four months after the operation. **e** Satisfactory knee motor function

Our study found no significant differences among the three groups in the time from injury to surgery (*P* = 0.057), duration of surgery, (*P* = 0.235), WOMAC score (*P* = 0.770), or overall complications (*P* = 0.147). There may be a significant difference in the number of days in the hospital (*P* = 0.004), HSS score (*P* < 0.001), and lateral knee pain and lateral hamstring tightness (*P* < 0.001). In a comparison of the three groups, there was a significant increase in the length of hospital stay for patients with TPFs that involved both the proximal fibula and PJF (group A vs. group C, *P* = 0.038), and only fractures of the proximal fibula also increased the length of hospital stay (group B vs. group C, *P* = 0.013). There was no significant increase in the length of hospital stay for patients with PJF fractures alone (group A vs. group B, *P* > 0.999). No significant differences were found in the WOMAC score among the three groups (*P* = 0.770). Jiang et al. examined 27 knee specimens and found that the bone density of the tibial plateau, but not the proximal fibula, decreased with age. Nonuniform settlement of the tibial plateau after weight-bearing is closely related to medial knee osteoarthritis [14]. However, no studies on the proximal fibula and traumatic osteoarthritis in patients with TPFs were available. We think that TPFs have no significant effect on the tendency to develop early posttraumatic osteoarthritis. Patients with proximal fibular fractures had a significantly lower HSS score than those in the other two groups (group A vs. group C, *P* < 0.001; group B vs. group C, *P* = 0.036), and a PJF fracture had no significant effect on the HSS score (group A vs. group B, *P* = 0.429). Therefore, early knee function is poorer in patients with proximal fibular fractures than in patients with an intact proximal fibula (Figs. 2, 3, 4). Patients with proximal fibular fractures had a significantly higher incidence of lateral knee pain and lateral hamstring tightness (group A vs. group C, *P* < 0.001; group B vs. group C, *P* < 0.001), which agrees with the results of another study.

Proximal fibular fractures have adverse effects on recovery in patients with TPFs, increasing the length of hospital stay, decreasing knee function, and causing lateral knee pain and lateral hamstring tightness. Bozkurt et al. first classified TPFs combined with a proximal fibular fracture in 2005 [15]. In 2018, the concept of the fibular column was first mentioned, and six new injury types were defined [26]. The role of the proximal fibula in TPFs has received increasing attention from clinicians. In a comparison of 55 patients with TPFs, Bozkurt et al. found that the Rasmussen score of patients with an intact proximal fibula was significantly better than that of patients with proximal fibular fractures. In addition, regardless of how the proximal fibular fracture is treated, almost all patients present with lateral knee pain and lateral hamstring tightness, which is not found in patients with an intact proximal fibula [15]. We have also observed this problem in patients with Schatzker type VI TPFs, and conservative treatment of proximal fibular fractures generally results in varying degrees of lateral knee pain and lateral hamstring tightness. We believe that this discomfort may not be related to the fibula itself but may be due to the involvement of the PLC at the same time as the fracture of the proximal fibula and subsequent adhesion and chronic pain symptoms. Whether proximal fibular fractures should be treated surgically is highly controversial, and pain can limit patients' ability to perform knee exercises, leading to poor results in terms of the HSS score. We believe that surgical treatment of proximal fibular fractures should be considered in patients with TPFs, not only to restore stability to the PLC of the knee but also to allow for early passive movement of the knee. At the same time, to avoid complaints of lateral knee pain and lateral hamstring tightness, for patients with proximal fibular fractures, the relevant examinations should be improved to exclude injury to the PLC and determine whether the PLC still shows long-term pathological changes after fracture healing.

The PJF is an important part of the PTFJ, which protects the proximal end of the fibula. When a PJF fracture occurs, the trauma is transmitted to the proximal fibula via the PTFJ, suggesting a very violent injury to the tibial plateau. Although Chang et al. found that a PJF fracture was an influencing factor for TPFs combined with a proximal fibular fracture [16], we did not find that PJF had a significant influence on the length of hospital stay, duration of surgery, postoperative knee function, or lateral knee pain and lateral hamstring tightness.

There are some limitations to this study. First, it was a single-centre study with a small sample size, and the results may demonstrate a regional bias. It is necessary to conduct multicentre research with large sample sizes. Second, the follow-up time in our study was short, and studies with a longer follow-up period are needed to determine the effect of proximal fibular fractures on TPFs.

## Conclusion

Overall, early functional recovery in patients with Schatzker type VI TPFs and combined proximal fibular fracture is more decisive than PJF involvement for prognosis.

## Data Availability

The datasets used and/or analyzed during the current study are available from the corresponding author on reasonable request.
